# Comparative genomics of multidrug-resistant *Enterococcus spp*. isolated from wastewater treatment plants

**DOI:** 10.1186/s12866-019-1683-4

**Published:** 2020-01-24

**Authors:** Haley Sanderson, Rodrigo Ortega-Polo, Rahat Zaheer, Noriko Goji, Kingsley K. Amoako, R. Stephen Brown, Anna Majury, Steven N. Liss, Tim A. McAllister

**Affiliations:** 1 Agriculture and AgriFood Canada, Lethbridge Research and Development Center, 5403 1 Avenue South, PO Box 3000, Lethbridge, T1J 4B1 Canada; 2 School of Environmental Studies, Queen’s University, Kingston, K7L 3N6 Canada; 3 Canadian Food Inspection Agency, National Centre for Animal Disease, Lethbridge Laboratory, Lethbridge, T1J 3Z4 Canada; 4 Department of Chemistry, Queen’s University, Kingston, K7L 3N6 Canada; 5 Public Health Ontario, Kingston, K7L 3K3 Canada; 6 Department of Biology, Ryerson University, Toronto, M5B 2K3 Canada

**Keywords:** Vancomycin resistant Enterococcus (VRE), Enterococci, Genomics, Antimicrobial resistance, Mobilome, Genome size, Wastewater, Pangenome, Biomarker

## Abstract

**Background:**

Wastewater treatment plants (WWTPs) are considered hotspots for the environmental dissemination of antimicrobial resistance (AMR) determinants. Vancomycin-Resistant *Enterococcus* (VRE) are candidates for gauging the degree of AMR bacteria in wastewater. *Enterococcus faecalis* and *Enterococcus faecium* are recognized indicators of fecal contamination in water. Comparative genomics of enterococci isolated from conventional activated sludge (CAS) and biological aerated filter (BAF) WWTPs was conducted.

**Results:**

VRE isolates, including *E. faecalis* (*n* = 24), *E. faecium* (*n* = 11), *E. casseliflavus* (n = 2) and *E. gallinarum* (n = 2) were selected for sequencing based on WWTP source, species and AMR phenotype. The pangenomes of *E. faecium* and *E. faecalis* were both open. The genomic fraction related to the mobilome was positively correlated with genome size in *E. faecium* (*p* < 0.001) and *E. faecalis* (*p* < 0.001) and with the number of AMR genes in *E. faecium* (*p* = 0.005). Genes conferring vancomycin resistance, including *van*A and *van*M (*E. faecium*), *van*G (*E. faecalis*), and *van*C (*E. casseliflavus*/*E. gallinarum*), were detected in 20 genomes. The most prominent functional AMR genes were efflux pumps and transporters. A minimum of 16, 6, 5 and 3 virulence genes were detected in *E. faecium*, *E. faecalis*, *E. casseliflavus* and *E. gallinarum,* respectively. Virulence genes were more common in *E. faecalis* and *E. faecium*, than *E. casseliflavus* and *E. gallinarum*. A number of mobile genetic elements were shared among species. Functional CRISPR/Cas arrays were detected in 13 *E. faecalis* genomes, with all but one also containing a prophage. The lack of a functional CRISPR/Cas arrays was associated with multi-drug resistance in *E. faecium*. Phylogenetic analysis demonstrated differential clustering of isolates based on original source but not WWTP. Genes related to phage and CRISPR/Cas arrays could potentially serve as environmental biomarkers.

**Conclusions:**

There was no discernible difference between enterococcal genomes from the CAS and BAF WWTPs. *E. faecalis* and *E. faecium* have smaller genomes and harbor more virulence, AMR, and mobile genetic elements than other *Enterococcus spp*.

## Background

Enterococci are ubiquitous in nature and can be found in a variety of environments, including soil, plants, surface water, wastewater, food, and the gastrointestinal tract of animals and humans [43, 60]. *Enterococcus faecalis* and *Enterococcus faecium*, are associated with a variety of clinical infections of the urinary tract, heart, surgical wounds, bloodstream and neonates [67] as well as indicators of fecal contamination [10]. The ability to treat infections caused by *Enterococcus spp*. is hindered by the development and spread of antimicrobial resistance (AMR) [1]. Resistance to antimicrobials of last resort, such as vancomycin, impairs the control of enterococcal infections and is usually accompanied by resistance to other antimicrobials [24, 32].

Enterococci and antimicrobials are excreted in urine and feces, and in urbanized developed nations, most of this waste is transported to and treated in wastewater treatment plants (WWTPs) prior to discharge into surface waters. WWTPs could be considered points of control for the environmental dissemination of AMR and ideal environments to investigate the epidemiology of AMR from a “One Health” perspective [2, 44, 57]. Within this environment, enterococci can not only exchange genes coding for AMR, but also for heavy metal resistance as well as other genes that increase persistence and survival in other environments [3]. This outcome can facilitate the broader dissemination of AMR genes [2]. Comparative genomics has been applied to identify genes responsible for virulence, AMR, metabolism, secondary metabolite production and gene mobility. Comparative genomics can also be used to compare genes from other functional categories, to predict the ecological fitness of strains, and to discern evolutionary relationships among species.

We previously isolated a number of species of enterococci from two WWTPs with different treatment processes, a conventional activated sludge (CAS) and a biological aerated filter (BAF) system, with *E. faecalis* being the dominant species identified [61]. This work demonstrated changes in AMR phenotypes between wastewater enterococci before and after treatment and between WWTPs. In the current study, we selected 39 wastewater enterococci for sequencing out of 1111 enterococci isolated, including 308 that exhibited vancomycin resistance in broth culture. Isolates were selected so as to be representative of before and after treatment in both WWTPs [61]. We hypothesized that the genomes would not cluster by treatment process but genomes from the BAF system may contain more biofilm-related genes than those from the CAS system. We also proposed that there would be more virulence, AMR, and genetic mobility genes in *E. faecalis* and *E. faecium* than other *Enterococcus spp*. and that the larger genomes in these clinically relevant species would correlate with the number of mobile genetic elements and genes conferring fitness for survival in a broader range of environments.

## Results

### Sequence statistics and Pan-genomic analysis

A summary of sequencing statistics for the 39 *Enterococcus spp*. genomes can be found in Table 1. The genomes ranged from 2.48–3.54 Mbp. The genomes of *E. casseliflavus* and *E. gallinarum* (3.37–3.54 Mbp, 3161–3344 genes) were larger than those of *E. faecalis* (2.69–3.09 Mbp, 2528–3051 genes) and *E. faecium* (2.48–3.02 Mbp, 2374–2992 genes). The GC content of the genomes ranged from 37.3–37.7%, 37.5–38.1%, and 40.4–42.9% for *E. faecalis*, *E. faecium*, and *E. casseliflavus*/*E. gallinarum*, respectively.

**Table 1:** Genome Characteristics of *Enterococcus spp*. Isolated from Municipal Wastewater Treatment Plants

Strain	Location	Species	# of Contigs	Size (bp)	%GC	Genes	CDSs	ST*
B72	BAF FE	*E. casseliflavus*	32	3,538,396	42.8	3344	3283	NA
B79	BAF FE	*E. casseliflavus*	49	3,527,325	42.9	3327	3268	NA
W41	CAS PE	*E. faecalis*	21	2,693,209	37.7	2528	2471	116
B139	BAF FE	*E. faecalis*	30	2,720,730	37.7	2553	2496	138/501
W314	CAS PE	*E. faecalis*	11	2,721,427	37.6	2583	2524	277
C34	CAS PE	*E. faecalis*	13	2,731,087	37.6	2615	2556	715
R95	BAF PE	*E. faecalis*	38	2,761,310	37.6	2596	2538	674
W350	CAS FE	*E. faecalis*	28	2,789,796	37.6	2731	2673	84
R76	BAF FE	*E. faecalis*	51	2,800,339	37.6	2690	2627	16
C106	CAS PE	*E. faecalis*	24	2,817,683	37.4	2670	2610	16
B48	BAF PE	*E. faecalis*	29	2,822,491	37.5	2701	2641	16
B168	BAF PE	*E. faecalis*	39	2,834,215	37.5	2725	2667	21
W191	CAS FE	*E. faecalis*	22	2,839,739	37.6	2745	2684	207
W460	CAS FE	*E. faecalis*	27	2,848,194	37.4	2733	2674	672
H120S2	BAF PE	*E. faecalis*	21	2,853,021	37.4	2731	2670	16
C33	CAS PE	*E. faecalis*	19	2,860,595	37.3	2722	2662	16
R378	BAF FE	*E. faecalis*	50	2,892,126	37.5	2858	2796	326
W75	CAS FE	*E. faecalis*	59	2,901,424	37.5	2838	2776	209
B6	BAF FE	*E. faecalis*	48	2,906,126	37.5	2796	2738	26
R395	BAF PE	*E. faecalis*	35	2,951,239	37.5	2844	2785	40
W195	CAS FE	*E. faecalis*	30	2,970,793	37.5	2865	2806	40
H114S2	BAF PE	*E. faecalis*	35	2,979,979	37.4	2881	2822	40
C379	CAS FE	*E. faecalis*	42	2,988,783	37.5	2902	2844	40
R61	BAF FE	*E. faecalis*	37	3,004,659	37.3	2969	2907	16
B150	BAF FE	*E. faecalis*	41	3,012,117	37.3	2955	2893	16
W37	CAS PE	*E. faecalis*	68	3,088,982	37.3	3051	2990	768
C329	CAS FE	*E. faecium*	63	2,480,628	38.1	2374	2305	40
B466	BAF FE	*E. faecium*	71	2,553,406	38.1	2512	2443	672
C567	CAS FE	*E. faecium*	21	2,778,016	37.9	2714	2644	1216
C12d	CAS PE	*E. faecium*	169	2,879,332	37.7	2823	2755	18
H101S2	BAF PE	*E. faecium*	177	2,884,826	37.8	2836	2770	18
H123S2	BAF PE	*E. faecium*	170	2,912,775	37.7	2871	2803	18
H53S1	CAS PE	*E. faecium*	165	2,959,005	37.6	2915	2847	18
F11j	CAS PE	*E. faecium*	181	2,964,368	37.6	2916	2848	18
R407	BAF PE	*E. faecium*	176	3,005,175	37.5	2955	2887	18
B492	BAF FE	*E. faecium*	189	3,008,305	37.5	2961	2893	18
R337	BAF PE	*E. faecium*	195	3,023,784	37.5	2992	2924	18
W17	CAS FE	*E. gallinarum*	14	3,367,806	40.5	3161	3102	NA
G12 s	CAS PE	*E. gallinarum*	30	3,442,529	40.4	3303	3244	NA

BAF: biological aerated filter, CAS: conventional activated sludge, PE: primary effluent, FE: final effluent, % GC: guanine-cytosine content, CDS: protein coding sequence, ST: sequence type, NA: not available

The range in contigs generated during sequencing was greater in *E. faecium* (21–195 contigs) than in other species (11–68 contigs), likely due to the presence of repetitive and insertion genetic elements complicating assembly [54]. Genome sizes were greater for vancomycin and multi-drug resistant strains of *E. faecium* (3.04 Mbp) than for susceptible strains (2.60 Mbp). The genome size of vancomycin-resistant and multi-drug resistant *E. faecalis* was similar to their susceptible counterparts.

**Fig. 1:**
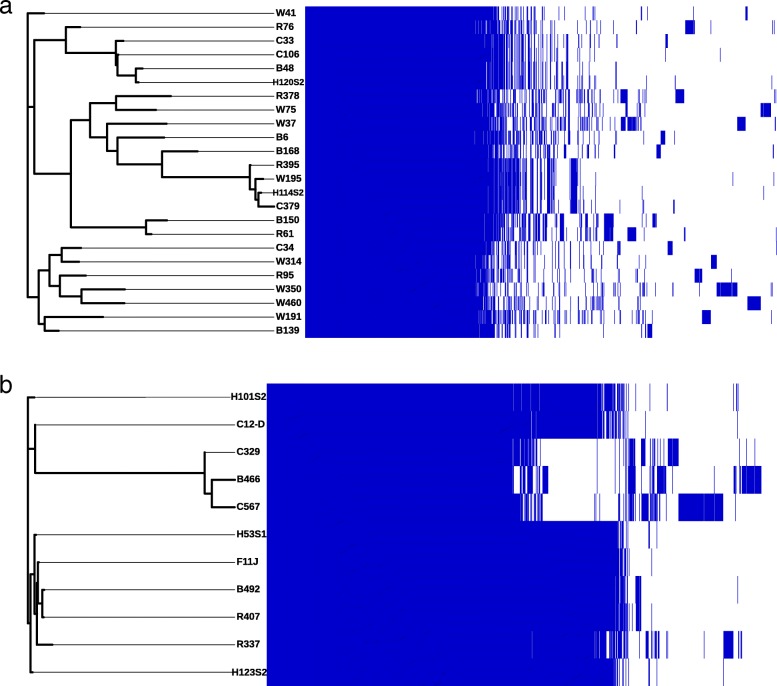
*Enterococcus faecalis (****a****) and Enterococcus faecium (****b****)* pan-genome illustrated as a matrix with the core SNP tree of the strains on the left and a presence (blue) and absence (white) matrix of core and accessory genes

The *E. faecalis* pangenome consisted of 5708 genes with a core of 2054 genes (36%), a soft-core of 91 genes (1.6%), a shell genome of 1193 (20.9%) and a cloud genome of 2370 genes (41.5%; Fig. 1 a). The *E. faecium* pangenome consisted of 3950 genes with a core of 1959 genes (49.6%), a shell of 1186 genes (30%) and a cloud genome of 805 genes (20.4%; Fig. 1 b).

### Multi-locus sequence typing

In the current study, 4 sequence types (STs) for *E. faecium* and 15 STs for *E. faecalis* were identified (Table 1). Eight *E. faecium* genomes belonged to ST18, part of the clonal complex 17 (CC-17). Out of the *E. faecalis* STs identified in this study, ST16 (*n* = 7) and ST40 (*n* = 4) were the most common.

### Phenotypic antimicrobial resistance profiles

Sequenced enterococci exhibited a number of phenotypic antimicrobial resistant profiles, with some isolates being resistant to as many as seven antimicrobials (Table 2). VAN^R^, TEC^R^, AMP^R^, ERY^R^ were among the most common resistant phenotypes found in enterococci.

**Table 2:** Phenotypic Antimicrobial Resistance Profiles of sequenced *Enterococcus spp* isolates

Isolate	AMR Phenotype*
*E. casseliflavus B72*	ERY^I^, Q-D^R^
*E. casseliflavus B79*	VAN^I^, ERY^I^, Q-D^I^
*E. faecalis W460*	ERY^I^, Q-D^R^
*E. faecalis W350*	DOX^R^, ERY^I^, LZD^I^
*E. faecalis W191*	DOX^R^, Q-D^R^
*E. faecalis H120S2*	ERY^R^, STR^I^
*E. faecalis H114S2*	VAN^R^, TEC^R^, AMP^R^, LVX^R^
*E. faecalis C34*	VAN^R^, TEC^R^, ERY^I^, LZD^I^, Q-D^R^
*E. faecalis C33*	TEC^R^, DOX^R^, ERY^R^, LZD^I^,LVX^R^, Q-D^R^
*E. faecalis B168*	DOX^I^, ERY^I^, LZD^I^, Q-D^R^
*E. faecalis B150*	TEC^R^, DOX^I^, ERY^R^, LZD^R^, Q-D^R^
*E. faecalis B6*	DOX^R^, ERY^I^, Q-D^R^
*E. faecalis B48*	DOX^R^, ERY^R^, LZD^R^, Q-D^R^
*E. faecalis B139*	VAN^I^, LZD^I^, Q-D^R^
*E. faecalis C106*	TEC^R^, DOX^R^, ERY^R^, GEN^R^, Q-D^R^
*E. faecalis C379*	DOX^R^, ERY^I^, Q-D^R^
*E. faecalis R61*	VAN^I^, TEC^R^, DOX^R^, ERY^R^, GEN^I^, LZD^I^, Q-D^R^
*E. faecalis R76*	DOX^R^, ERY^R^, GEN^R^, Q-D^R^, STR^R^
*E. faecalis R95*	DOX^I^, ERY^R^, GEN^I^, LZD^I^, Q-D^R^, STR^R^
*E. faecalis R378*	ERY^I^, Q-D^I^
*E. faecalis R395*	VAN^I^, TEC^R^, DOX^R^, ERY^I^, Q-D^R^
*E. faecalis W37*	DOX^I^, ERY^R^, LVX^R^, Q-D^I^, STR^R^
*E. faecalis W41*	VAN^I^, TEC^R^, DOX^R^, ERY^I^, Q-D^R^
*E. faecalis W75*	VAN^I^, Q-D^I^
*E. faecalis W195*	DOX^I^, ERY^I^, Q-D^R^
*E. faecalis W314*	TEC^R^, ERY^I^, LZD^R^, Q-D^R^
*E. faecium R407*	VAN^R^, TEC^R^, AMP^R^, ERY^R^, GEN^R^, LVX^R^, STR^R^
*E. faecium R337*	VAN^R^, TEC^R^, AMP^R^, ERY^R^, NIT^I^, LVX^R^, STR^R^
*E. faecium H53S1*	VAN^R^, TEC^R^, AMP^R^, ERY^R^, LVX^R^, STR^I^
*E. faecium F11 J*	VAN^R^, TEC^R^, AMP^R^, ERY^R^, LVX^R^, STR^R^
*E. faecium C329*	TEC^R^, ERY^I^, NIT^R^, LZD^I^, LVX^R^
*E. faecium B492*	VAN^R^, TEC^R^, AMP^R^, ERY^R^, LVX^R^, STR^R^
*E. faecium B466*	AMP^R^, DOX^R^, ERY^R^, NIT^I^, LVX^I^
*E. faecium C12D*	VAN^R^, TEC^R^, AMP^R^, ERY^I^, LVX^R^
*E. faecium C567*	ERY^R^, NIT^I^, LZD^I^
*E. faecium H101S2*	VAN^R^, TEC^R^, AMP^R^, ERY^R^, LVX^R^, STR^I^
*E. faecium H123S2*	VAN^R^, TEC^R^, AMP^R^, ERY^R^, LVX^R^, STR^I^
*E. gallinarum W17*	VAN^I^
E. gallinarum G12S	VAN^I^

a AMR phenotypic profiles using R for resistant to the antimicrobial and I for intermediately resistant to the antimicrobial. Antimicrobials used for disc susceptibility testing were vancomycin (VAN), teicoplanin (TEC), amipicillin (AMP), doxycycline (DOX), erythromycin (ERY), levofloxacin (LVX), linezolid (LZD), nitrofurantoin (NIT), gentamicin (GEN), streptomycin (STR), quinupristin/dalfopristin (Q-D), and tigecycline (TGC)

### Phylogeny

**Fig. 2:**
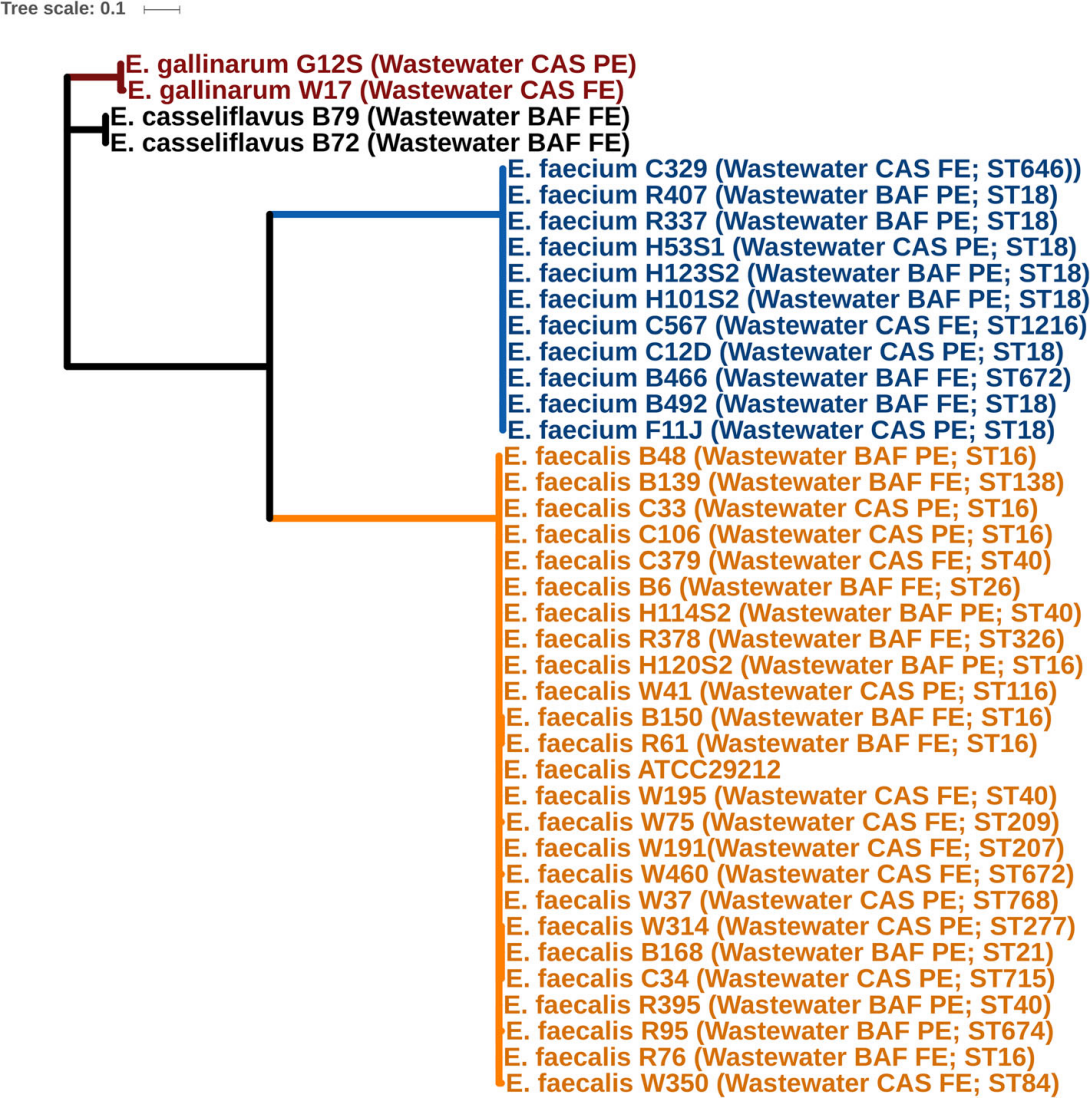
Phylogenetic Tree of all Enterococcus spp. isolated from wastewater using *Enterococcus faecalis* ATCC 29212 as the reference genome

**Fig. 3:**
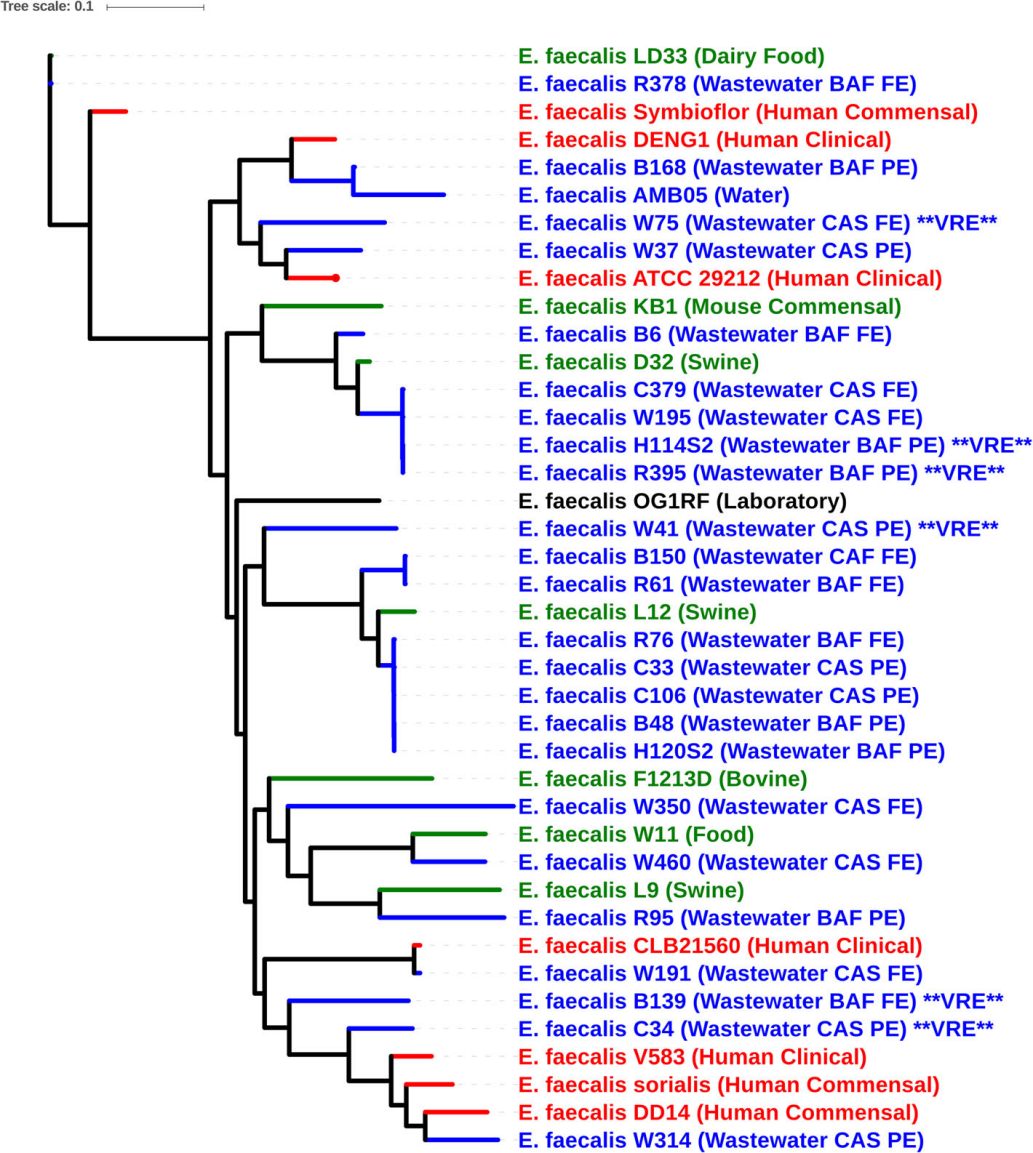
Phylogenetic tree of *Entercoccus faecalis* genome sequences from the present study and complete genome sequences from the NCBI GenBank database based on analysis of single-nucleotide variants (SNVs) of the core genes. *Enterococcus faecalis* ATCC29212 was used as the reference genome. Origin of Isolates are as indicated in the figures and are grouped by colour into clinical (red), agricultural/food (green) and wastewater/water (blue) groups

**Fig. 4:**
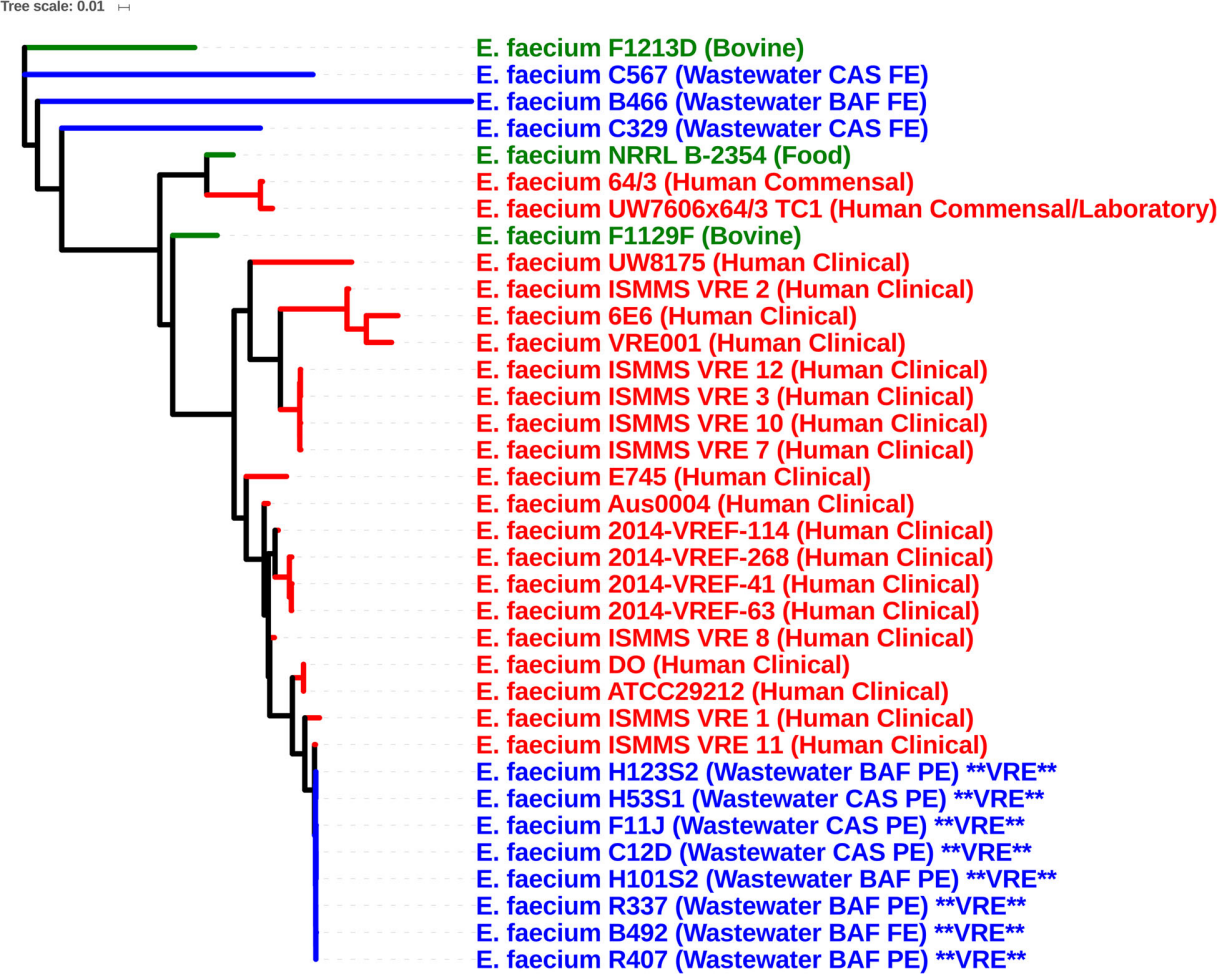
Phylogenetic tree of *Entercoccus faecium* genome sequences from the present study and genome sequences from the NCBI GenBank database based on analysis of single-nucleotide variants (SNVs) of the core genes. *Entercoccus faecium* DO served as the reference genome. Origin of isolates are as indicated in the figures and are grouped by colour into clinical (red), agricultural/food (green) and wastewater/water (blue) groups

Genomes did not cluster based on WWTP, but all species formed separate monophylogenetic groups (Fig. 2). The majority of wastewater *E. faecalis* isolates were more closely related to livestock and food-derived *E. faecalis* genomes, while seven wastewater strains (B139, B168, C34, W37, W75, W191, and W314) clustered with strains isolated from human infections (Fig. 3). None of the *E. faecalis* wastewater, human, and agriculture (and food-derived) isolates clustered together by source, suggesting that agricultural and human clinical strains are phylogenetically distinct. Vancomycin-resistant *E. faecalis* isolates also did not cluster as they belonged to different STs, unlike vancomycin-resistant *E. faecium*, which did cluster as all isolates belonged to CC-17 (Fig. 4). For *E. faecium*, wastewater strains clustered separately from most clinical strains (Fig. 4). The bovine strain *E. faecium* F1129F clustered with human clinical strains, whereas the other bovine strain, *E. faecium* F1213D did not. Three wastewater isolates (*E. faecium* C567, *E. faecium* B466, and *E. faecium* C329) were more closely related to *E. faecium* F1213D (bovine) *and E. faecium* NRRL B-2354 (food) than to clinical isolates.

### Clusters of orthologous groups (COGs): functional categories and genome size

Clusters of Orthologous Groups (COGs) are broad functional categories used to assign proteins to their specific function [69]. Functional categorization of proteins into different COGs revealed variation profiles among *Enterococcus spp*., but little difference among strains within species, with the exception of the mobilome and genes associated with energy production and conversion (Additional file 1, sheet 6). We assessed which functional categories of genes were disproportionately represented in the isolates collected from the WWTPs with expanded genomes.

**Fig. 5:**
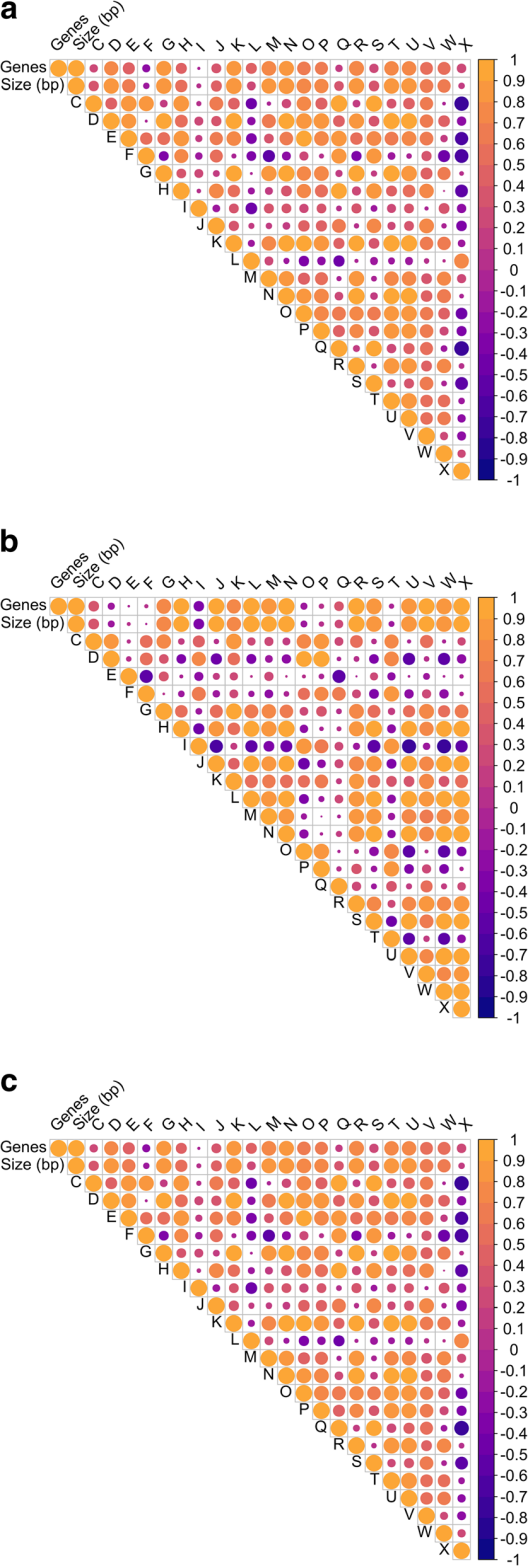
Correlation plots showing the correlations between different COG categories, genome size and number of genes in all of the pooled *Enterococcus* species (**a**), *E. faecalis,* (**b**) and *E. faecium* (**c**)

Given the variation in genome size between and within species, the relationships between genome size and the number of genes associated with specific functional categories was determined (Fig. 5; Additional file 1, Sheet 6). There were more COGs assigned to carbohydrate transport and metabolism, transcription, cell motility, secondary metabolite biosynthesis, transport, catabolism and signal transduction mechanisms in *E. casseliflavus* and *E. gallinarum* compared to enterococci more frequently associated with clinical infections.

When all of the wastewater *Enterococcus* genomes were pooled, there was a strong negative correlation (*p* < 0.001) between genome size and nucleotide transport and metabolism, lipid metabolism and translation, ribosomal structure and biogenesis and a strong positive correlation (*p* < 0.001) between genome size and cell motility (Fig. 5 a; Additional file 1, sheet 6). The total number of genes related to cell motility, signal transduction, and carbohydrate transport and metabolism were positively correlated (*p* < 0.001) with genome size. This is reflective of the greater genome size of environmental species compared to *E. faecium* and *E. faecalis*. The total number of genes related to cell division and chromosome partitioning, cell envelope biogenesis, outer membrane and post translational modification, protein turnover, and transcription were negatively correlated (*p* < 0.001) with genome size.

The species-specific patterns in genomic proportions for each functional category differ from the pooled genomes for the genus. In both *E. faecalis* and *E. faecium*, a larger genome was strongly correlated with the mobilome (*p* < 0.001) (Fig. 5 b and c), a functional category not included in the analysis of Konstantinidis and Tiedje [34]. In contrast, the mobilome was not correlated with genome size in the pooled *Enterococcus* genomes. There was also a positive correlation (*p* = 0.005) between the number of unique AMR genes and genome size of *E. faecium,* suggesting the acquisition of AMR genes occurs through horizontal gene transfer. For example, *E. faecium* R337 had a genome of 3.02 kbp, 58 genes associated with the mobilome and 23 AMR genes; while *E. faecium C329* had a genome of 2.48kbp and 15 genes associated with the mobilome and 3 AMR genes.

The total number of genes related to cell motility (*p* < 0.001), DNA replication, recombination, and repair (p < 0.001), extracellular structures (*p* < 0.001), and mobilome (*p* < 0.001) was positively correlated with genome size in *E. faecium*. The number of AMR genes also showed a positive correlation (*p* = 0.002) with the amount of genes related to the mobilome in this species (Fig. 5 c). The eight *E. faecium* genomes belonged to the same sequence type (CC-17), while *E. faecalis* genomes were more diverse.

### Antimicrobial resistance genes

In this study, we screened 39 multi-antimicrobial resistant enterococci genomes against the CARD database for antimicrobial resistance genes (ARGs) (Additional file 1, Sheet 8) and ten genes (*eatAv*, *eme*A, *lsa*A, *efr*A, *efr*B, *tet*L, *efm*A, *msr*C, *erm*Y, and *lsa*E) associated with multi-drug efflux pumps and other transporters were detected. These efflux proteins may confer intermediate resistance to a variety of antimicrobials.

Genes conferring glycopeptide (vancomycin and teicoplanin) resistance were detected in 20 of the genomes. In *E. faecium* and *E. faecalis*, resistance was conferred by *van*A and *van*M in *E. faecium* or *van*G in *E. faecalis*. Vancomycin resistance was mediated by *van*C, and this was the only ARG detected, in *E. casseliflavus* and *E. gallinarum.*

*Erm*B confers resistance to macrolide-lincosamide-streptogramin B (MLSB) antimicrobials and was found in *E. faecium* (*n* = 7) and *E. faecalis* (*n* = 4). Other *erm* genes (*erm*C, *erm*G, *erm*T, *erm*Y) were detected in one *E. faecium* genome. *msr*C, which codes for a macrolide efflux pump, was only detected in *E. faecium* (*n* = 11). The most common macrolide resistance gene detected in enterococcal genomes was *erm*B (*n* = 15).

Thirteen of the enterococci isolates were resistant to high concentrations of gentamicin and streptomycin. In our study, cross-resistance to levofloxacin and the aminoglycosides (gentamicin and streptomycin) occurred in 5 isolates with 3 additional isolates exhibiting intermediate resistance to one or more of these antimicrobials. In our study, additional aminoglycoside genes (*ant*(9′)-Ia, *aad*(6′), *aph*(3′)-*IIIa*, *SAT*-4, *ant*(6′)-*Ia*, and *aac*(6′)-*Ie*-*aph*(2″)-*Ia*) were detected in the genomes of up to 5 *E. faecalis* and 7 *E. faecium* aminoglycoside resistant isolates. Gentamicin resistance arises as the result of the acquisition of *aac*(6′)-*Ie*-*aph*(2″)-*Ia,* which was detected in 7 genomes (2 *E. faecalis* and 5 *E. faecium*) and confers resistance to all aminoglycosides except streptomycin [42]. The prevalence of streptomycin resistance versus gentamicin resistance differed between species, with streptomycin resistance being more common in *E. faecium* and gentamicin resistance more common in *E. faecalis*.

Genes encoding tetracycline resistance were detected in 26 of the genomes, including *E. faecium* and *E. faecalis*. In this study, determinants for macrolide and tetracycline were detected together in 16 of the enterococcal genomes. Genes associated with resistance to antimicrobials not included in the disc susceptibility panel were also detected. A gene associated with chloramphenicol resistance, *cat*, was detected in two *E. faecalis* genomes. Genes associated with diaminopyrimidine resistance (*dfr*E, *dfr*F, and *dfr*G) were also detected in *E. faecium* and *E. faecalis*. Two *E. faecalis* genomes also had genes associated with lincosamide resistance (*Inu*B and *Inu*G).

### Virulence genes

The number of shared virulence genes among genomes of the same species were 16, 6, 5 and 3 for *E. faecium*, *E. casseliflavus*, *E. faecalis*, and *E. gallinarum*, respectively (Additional file 1, Sheet 9–11). All of the *E. faecium* isolates contained genes related to adhesion to surfaces (*tuf*, *aga*, *efa*A, and *sgr*A), cell wall biosynthesis (phosphatase cytidylyltransferase, *upp*S), cellular defense (*lis*R), biofilm formation and surface proteins (*acm*, *esp*, *scm* and type A and B pili). Other functions including bile salt degradation (*bsh*), proteases (*tip/ropA),* biofilm formation (*bop*D), enolase (*eno)*, and antiphagocytosis and capsule formation (*rfb*A-1) were also identified. All of the *E. faecalis* genomes contained genes for cell adhesion (*tuf*), carbohydrate metabolism (*hyl*), endocarditic and biofilm association (ebp) pili (*ebp*A), Type III secretion proteins (*bop*D) and fibrinogen-binding proteins (*fss*1). All of the *E. casseliflavus* genomes contained the same five virulence genes with functions of: capsule biosynthesis (*cap*E), enolase (*eno*), leucine aminopeptidase (*lap*), heat shock protein (*hsp*60), and protein modification (*lpl*A1). All of the *E. gallinarum* genomes had an enolase (*eno*), a flagellar biosynthesis protein (*flh*A) and a bile salt hydrolase (*bsh*). One of the *E. gallinarum* genomes also contained genes related to capsule proteins and another isolated from effluent possessed 2 genes associated with metal transporter (*ssa*B and *psa*A) as well as those associated with the CAS system. Hyaluronidase (*hyl*) genes were detected in all the *E. faecalis* genomes.

### Mobile gene elements

ICE and transposons present in the genomes were identified and described using the ICEberg database (Table 3; Additional file 1, sheet 17). The transposon, Tn917 was identified in 8 of the sequenced *E. faecalis* genomes. One transposon, Tn6098 was present in all genomes. A multidrug resistance transposon, Tn5385 was also found in all *E. faecalis* genomes. Other Tn5801 and Tn6013-like ICE elements of unknown function were also present in all *E. faecium* isolates, in addition to a cadmium and arsenic resistance ICE, ICESde3396. All of the *E. gallinarum* and *E. casseliflavus* isolates had Tn916-type transposons (Tn6079, Tn6087 and Tn6084, respectively). Seven out of the unique 27 ICE were present in genomes of more than one *Enterococcus* species.

**Table 3:** Integrative conjugative elements (ICE) and transposons identified in the wastewater *Enterococcus spp.* genomes (*n* = 39)

Species	Common ICE	Function	Other Notable ICE	Function
*E. casseliflavus*	Tn6098 Tn6084 Tn6000(EfcTn1)	α-galactoside metabolism Tetracycline resistance Tetracycline resistance	No other ICE detected	
*E. faecalis*	Tn6098 Tn5385	α-galactoside metabolism Erythromycin, gentamicin, streptomycin, tetracycline, penicillin/β-lactam, mercury resistance	Tn917 Tn2008 Tn1545 ICESp23FST81 Tn6009 ICESde3396 Tn5301 Tn5276 ICESt1	Tetracycline resistance Chloramphenicol, erythromycin, streptomycin, kanamycin resistance Sulfamethoxazole, trimethoprim, chloramphenicol, erythromycin, streptomycin resistance Tetracycline, chloramphenicol resistance, toxin-antitoxin system Mercury resistance Kanamycin, arsenic and cadmium resistance Nisin biosynthesis Nisin biosynthesis, sucrose fermentation Type II restriction modification system
*E. faecium*	Tn6098 Tn5801 Tn6084 Tn6000(EfcTn1) ICESauTW20–2 ICESauT0131–2 ICESauJKD6008–2 ICESpsED99–1 ICESauMu3–1	α-galactoside metabolism Tetracycline resistance Tetracycline resistance Tetracycline resistance Unknown Unknown Unknown Unknown Unknown	Tn2008 Tn1545 ICESde3396 ICESt1 ICEAusCo10a-1 10,750-RD.2	See above See above See above See above Toxin-antitoxin system Type II restriction modification, spectinomycin, erythromycin resistance
*E. gallinarum*	Tn6098 Tn6079 Tn6087 ICEsde3396	α-galactoside metabolism Tetracycline, erythromycin resistance Tetracycline, antiseptic and antimicrobial resistance (unspecified) Kanamycin, arsenic and cadmium resistance	Tn2008 Tn1545	See above See above

### CRISPR-Cas arrays and bacteriophage

**Fig. 6:**
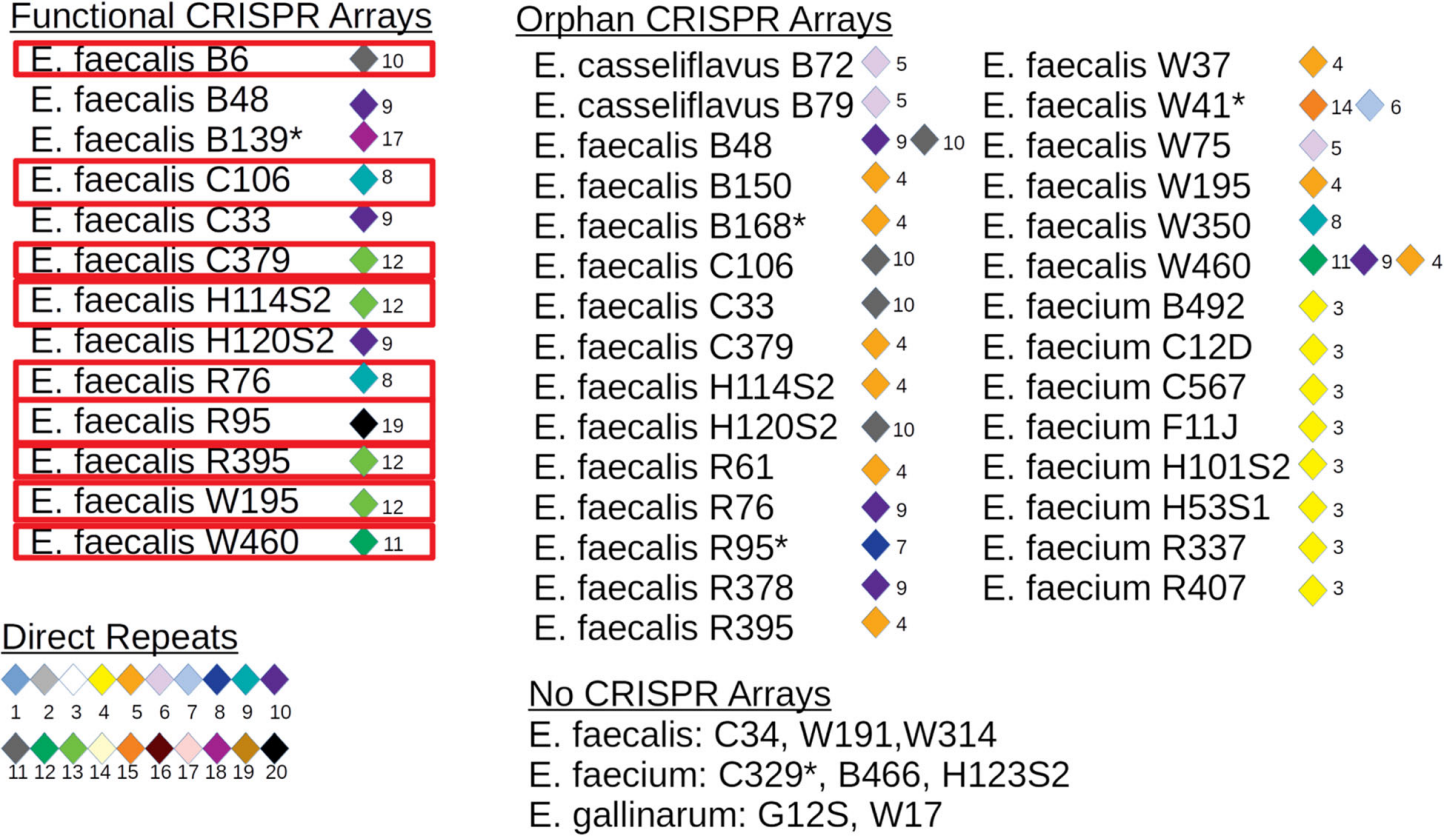
Pictorial of CRISPR-Cas arrays identified in the analysis of wastewater *Enterococcus spp* (*n* = 39) genomes. **a** Functional CRISPR arrays with the organization of direct repeats (diamonds) and spacers (numbers). **b** Orphan CRISPR arrays direct repeat and spacer organization. **c** Legend for numbered direct repeats and the genomes with no CRISPR arrays. The outlined genomes are those that contained both functional CRISPR arrays and prophage. The (*) represents genomes that contained no prophage

Type II CRISPR-Cas systems were detected in 13 *E. faecalis* genomes (Fig. 6). Orphan CRISPR arrays (without Cas genes) were identified in 27 of the genomes (Fig. 6). Comparison of CRISPR arrays flanked by Cas genes revealed unique arrays among *Enterococcus* species, but some arrays were shared among strains of the same species. Arrays identified in the sequenced *Enterococcus* genomes contained 4 to 20 direct repeat sequences associated with functional CRISPR arrays. An additional 72 unique spacers associated with orphan CRISPR arrays were identified in this study. Eleven *E. faecalis* and 10 *E. faecium* genomes lacked CRISPR-Cas systems. Any genomes lacking functional arrays exhibited resistance to 4 or more antimicrobial agents.

Functional CRISPR arrays and intact prophage were identified in 10 *E. faecalis* genomes, but the combination was not seen in the other 29 genome sequenced in this study. Some of the spacer regions identified in CRISPR arrays were 100% identical to incomplete prophage sequences, but these genomes still contained at least one prophage.

Bacteriophage-mediated transduction of AMR has been demonstrated in enterococci and potential virulence determinants have been identified in phage associated with *E. faecalis*. Phages found in the genomes were members of the Siphoviridae and Myoviridae (Additional file 1, Sheet 12). Thirty-four of the 39 genomes contained at least one putative phage ranging in size from 19.2 kb to 70.6 kb. A total of 55 unique intact prophages were identified across 34 sequenced genomes. *E. faecium* and *E. faecalis* contained up to 3 intact prophages, whereas E. casseliflavus and E. gallinarum contained 1 or 2 intact prophages.

### Secondary metabolites

Bacteriocins were identified in 8 *E. faecalis* and 9 *E. faecium* genomes in addition to 1 *E. gallinarum* genome (Additional file 1, Sheet 18). Enterocin A was identified in nine *E. faecium* genomes. Lantipeptides were identified in 3 *E. faecalis* genomes as cytolysins, which have both haemolytic and bacteriolytic activities [12]. Lassopeptides were identified in 6 *E. faecalis* genomes. Terpenes were detected in all *E. casseliflavus* and *E. gallinarum,* but not in *E. faecalis* or *E. faecium* genomes. Aryl polyene was detected in one *E. faecalis* (C34) genome.

### Biomarker search

The small number of genomes limited the identification of biomarkers, particularly for searches within the same species isolated from different sources (Additional file 1, Sheet 19). These biomarkers are genes or gene fragments only present in one group of genomes and not others making them possible identifiers of the origin of collected isolates. The majority of searches have identified biomarkers with scores below a correlation cut-off of 0.95. However, in our study, *E. faecalis* from wastewater that clustered with agricultural and animal sources revealed a biomarker associated with CRISPR-associated genes that differentiated (score = 0.8043) these isolates from *E. faecalis* from wastewater that clustered with human sources. A comparison of *E. faecium* from clinical (inclusion) and wastewater (exclusion) sources yielded 7 biomarkers with scores greater than 0.80. These were associated with phage (*n* = 6) and hypothetical proteins (*n* = 1). A search for potential biomarkers that distinguished among species in this study resulted in 98 signatures for *E. faecalis*, 130 signatures for *E. faecium*, and 3 signatures *E. casseliflavus* and *E. gallinarum*. These signatures include genes related to various types of nucleotide and carbohydrate metabolism, as well as other functions.

## Discussion

### Sequence statistics and Pan-genomic analysis

There was considerable variation in the size of the genomes and the number of contigs generated by sequencing each genome. The variation in the size of the genomes within a species could be a result of differences in the size of the chromosome and the presence/absence of plasmids. The variation in the number of contigs is likely due to the presence of repetitive and insertion genetic elements complicating assembly [54]. While the number of genomes used to generate the pan-genome in our study was small, the pan-genome of *Enterococcus spp*. is considered open as it is continually expanding and acquiring new accessory genome elements from other enterococci and bacterial species [80].

### Multi-locus sequence typing

In *E. faecium*, CC-17 is associated with clinical infections and has been detected in treated and untreated wastewater, [13] suggesting that the majority of *E. faecium* sequenced from wastewater originated from humans. In *E. faecalis*, ST16 and ST40 have previously been associated with high level gentamicin resistance in clinical isolates and in isolates from pigs [24, 59]. However, high level gentamicin resistance was not found in any *E. faecalis* with these sequence types. However, only 5 of the isolates in this study (4 *E. faecalis* and 1 *E. faecium*) exhibited high level gentamicin resistance. The association of these sequence types and gentamicin resistance may differ between studies because of geographical location, as gentamicin resistance is transferable, and because it may not be present in all ST16 and ST40 *E. faecalis* isolates.

### Phylogeny

The genomes forming monophylogenetic groups support our previous results of speciation of enterococci based on the *gro*ESL locus [61, 79]. The diversity of wastewater strains maybe a reflection of their origin from clinical, companion animal or agricultural sources. There was more genetic diversity in vancomycin-resistant *E. faecalis* than *E. faecium*. The distinct clustering between clinical and wastewater strains of *E. faecium* may be due to the large accessory genome and characterization of these genes may provide insight into the mechanisms whereby enterococci adapt to specific environments.

A disproportionate increase in genes associated with energy conversion, regulatory function, transport and secondary metabolism has been noted with expansion in genome size in previous comparative bacterial genomic studies [6, 34, 66]. So, an analysis of the COGs that are over represented in the expanded genomes of *E. faecalis* and *E. faecium* was completed to determine if some of these COGs could be increasing the fitness of multi-drug resistant enterococci. This could ultimately increase the risk of infection with these strains and the transfer of virulence and AMR determinants to other bacteria.

In *E. casseliflavus* and *E. gallinarum* some COGs were over represented (i.e., carbohydrate transport and metabolism, transcription, cell motility, secondary metabolite biosynthesis, transport, catabolism and signal transduction mechanisms). These functional categories could allow for higher fitness in aquatic environments where more diverse substrates are typically available at much lower concentrations than in the digestive tract. The increase in cell motility related genes may also enable these species to undertake chemotaxis in aquatic environments where nutrients may be scarce [58]. Compared to *E. faecalis* and *E. faecium*, these genomes also contained more genes encoding for secondary metabolites including antimicrobial agents. Although these genes are not required for growth, they can confer competitiveness in diverse environments [31]. *E. casseliflavus* and *E. gallinarum* are known to be more environmentally fit than *E. faecalis* and *E. faecium* as a result of a variety of mechanisms. For instance, the yellow pigment of *E. casseliflavus* can protect this species from photo-inactivation in aquatic environments [36]. However, *E. faecium* and *E. faecalis* are still the predominant species in wastewater, likely due to the continuous input of fecal waste into these systems.

The number of genes related to the mobilome increased with genome size in *E. faecium* and *E. faecalis* and this would suggest that the mobilome is a significant factor in the evolution of these bacteria within wastewater, contributing to genomic expansion and diversity. However, there was a lack of diversity in *E. faecium* isolates compared to *E. faecalis,* suggesting that *E. faecium* isolates may be more specifically adapted to clinical environments.

### Antimicrobial resistance genes

Vancomycin-resistant enterococci have been known to exhibit resistance to a number of antimicrobials [32, 74]. Enterococci are also intrinsically resistant to beta-lactams, aminoglycosides and streptogramins and can acquire antimicrobial resistance through horizontal gene transfer [32, 42, 74]. There are a variety of ARGs that confer vancomycin resistance, with *van*A, *van*B and *van*C being the most common in wastewater enterococci. The most common determinant for teicoplanin resistance is *van*Z, which can be integrated into the van operon, although it is absent in the *van*B operon, and confers resistance to both vancomycin and teicoplanin [19]. As a result, teicoplanin resistance is commonly associated with vancomycin resistance. Although rarely, teicoplanin resistance without vancomycin resistance is likely due to changes in the promoter of the van operon or due to the presence of a different resistance mechanism [14, 21, 35].

Resistance to erythromycin and other macrolides can arise as a result of mutations in the 23S rRNA gene or by efflux pumps [42]. Macrolides are used extensively in both humans and animals. Blanch et al. [9] observed that most wastewater isolates with high-level vancomycin resistance were also resistant to erythromycin, suggesting that erythromycin resistance may favour the persistence of VRE in the environment. The modification of the 23S rRNA target by methylase genes, like *erm*B, can also confer resistance to streptogramins [42].

Enterococci exhibit intrinsic resistance to low concentrations of aminoglycosides as a result of the presence of *aac*(6′)-*Ii*. Gentamicin and streptomycin are clinically-important as they are not inactivated by *aac*(6′)-*Ii*; and *E. faecium* are typically sensitive to these antimicrobials [42]. Aside from cross-resistance to other antimicrobial classes, like fluoroquinolones, resistance to these aminoglycosides is likely acquired. Others have shown that aminoglycoside resistance genes are frequently encoded on plasmids and transposons [42]. Streptomycin resistance either involves the inhibition of the drug at the ribosomal level or enzyme inactivation by an acquired streptomycin adenyltransferase [42].

There are multiple tetracycline resistance genes. *Tet*(L) encodes an efflux protein and *tet*(M) and *tet*(S) encode for ribosomal protection proteins. Disk susceptibility testing revealed that these isolates were resistant to doxycycline, whilst those containing *tet*(L) were susceptible, suggesting specificity for the *tet*(L) efflux protein. In general, bacteria that are resistant to doxycycline are also resistant to tetracycline and oxytetracycline [26, 56]. Tetracycline resistance can be due to efflux pumps or ribosomal protection mechanisms, which can be chromosomal and/or plasmid-borne. Co-selection of tetracycline and macrolide resistance in environmental enterococci may occur [39, 40].

### Virulence genes

The virulence genes detected have additional functions for improved environmental fitness. For instance, the majority of the virulence genes detected in the genomes from this study were also associated with biofilm formation or adherence to surfaces (i.e., *ace*, *acm*, *agg*, *bop*, *ccf*, *cob*, *cpd*, *ebp*ABC, *ecb*A, *efa*A, *esp*, *fsr*ABC, *gel*E, *pil*, *scm*, *sgr*A, *spr*E, and *srt*). These genes are ubiquitous as they likely play a role in the fitness of enterococci in both the human digestive tract and WWTPs. A number of capsule protein genes were also common among the genomes and not only confer resistance to phagocytosis in humans and animals [48, 50], but also to predation by amoeba and bacteriophage in aquatic environments [51, 73]. Hyaluronidase (*hyl*) genes have been associated with increased vancomycin resistance and virulence in mouse peritonitis models [50].

### Mobile genetic elements

Mobile genetic elements (MGEs) play an important role in horizontal gene transfer and the spread of AMR among isolates in the environment, humans and animal hosts. MGEs include plasmids, transposable elements, prophages and various genomic islands such as integrative conjugative elements (ICE) [71]. The transposon Tn917 is widely distributed in enterococci [64]. All of these strains exhibited erythromycin resistance and *erm*(B) was found to be associated with Tn1545 and Tn917 [15]. Transposon Tn6098 was in all of the genomes and possessed genes associated with α-galactoside metabolism. Transposon Tn5385 was found in all of the *E. faecalis* with these isolates exhibiting erythromycin and doxycycline resistance as this transposon commonly carries these resistance genes [53]. Tn916-type transposons found in *E. casseliflavus* and *E. gallinarum* can carry genes coding for tetracycline, minocycline and erythromycin resistance [52, 55]. While these transposons were detected in *E. casseliflavus* and *E. gallinarum,* they did not exhibit erythromycin resistance and no associated AMR genes were detected in their genomes.

### CRISPR-Cas arrays and bacteriophage

Type II CRISPR-Cas systems are typically described in enterococci. Multiple CRISPR arrays can often be detected in bacterial genomes, but not all arrays are accompanied by Cas genes. The absence of CRISPR/Cas systems may compromise genome defence, increasing the likelihood of acquisition of AMR determinants from bacteriophage and plasmids [47]. When a phage infects a bacterium, it incorporates spacers into the array within the bacterial chromosome and occasionally plasmids. The spacers are expressed as CRISPR RNAs (crRNAs) and provide a surveillance mechanism for descendant cells and guide the CRISPR/Cas system to enable cleavage of the protospacer sequence in the phage genome. The cleaved phage genomes are then cannibalized and can no longer support productive phage infection [5, 68]. CRISPR-Cas systems impact the evolution of both bacteria and phage populations. Transduction dependent horizontal gene transfer is a key driver of bacterial evolution and rapid viral evolution to evade CRISPR-Cas systems [68]. CRISPR/Cas arrays can also provide a record of previous and continued interaction between particular bacteria and phage [5, 65]. Spacers may limit the type of phage that can integrate into the genome, but bacteriophage can develop anti-CRISPR systems to promote their integration into the bacterial genome [11].

Phages found in the genomes were members of the Siphoviridae and Myoviridae. Other prophages in *Enterococcus spp*. belonging to Podoviridae, Inoviridae, Leviridae, Guttaviridae and Fuselloviridae have also been described [18, 41]. Prophages from the Siphoviridae family were the most prevalent across all species and are also commonly identified in lactic acid bacteria [72].

### Secondary metabolites

Bacteriocins are ribosomally synthesized antimicrobial peptides produced by Gram-positive and Gram-negative bacteria that have antimicrobial activity against closely related bacteria. They could provide a competitive advantage to the survival of bacteria in ecological niches that exhibit poor nutrient concentrations, heat and extreme pH [78]. Lantipeptides are also a growing class of bacteriocins with a large diversity of activity, structure, and biosynthetic machinery. Lantipeptides have multiple uses including as a limited class of antimicrobials [33]. Terpenes are most often associated with plants and fungi, and have been described in prokaryotes in only a few instances, including *Enterococcus spp* [7]. Terpenes can have a variety of functions including as antimicrobials, hormones, pigments, and flavor or odour constituents [45], but their role in *Enterococcus spp*. is unclear. Aryl polyene biosynthetic clusters produce a pigment that protects the organism from reactive oxygen species [62].

### Biomarker search

Biomarkers are genes or gene fragments only present in one group of genomes and not others making them possible identifiers of the origin of collected isolates. For instance, Weigand et al. [77] conducted a search within watershed and enteric enterococcal genomes and found shared phenotype and phylogeny between the two groups, but also identified several biomarkers for both sources. These biomarkers encoded accessory nutrient utilization pathways, including a nickel uptake operon and sugar utilization pathways including xylose were overrepresented in enteric genomes [77]. Genes that serve as biomarker for *E. casseliflavus* and *E. gallinarum* include genes related to various types of nucleotide and carbohydrate metabolism, and genes with other functions which can improve environmental fitness, including a variety of transporters and housekeeping genes related to DNA replication, transcription and translation.

## Conclusions

In this study, enterococci did not cluster phylogenetically based on point of isolation during wastewater treatment or on the type of WWTPs. Despite being the dominant species in wastewater, *E. faecalis* and *E. faecium* have smaller genomes and may be less equipped to survive outside of their target niche than *E. casseliflavus* and *E. gallinarum*. However, they do harbor more virulence, AMR, and mobile genetic elements than other *Enterococcus spp*. A larger genome size in *E. faecalis* and *E. faecium* was positively correlated with an expansion in the mobilome. In *E. faecium*, there was a positive correlation between the number of AMR genes and the mobilome. So, while the larger genome size of *E. casseliflavus* and *E. gallinarum* is accompanied by more genes related to metabolism and secondary functions, possibly increasing their fitness in the environment, this was not the case for *E. faecium* and *E. faecalis*. This study suggests that the key to understanding the impact of WWTPs on AMR dissemination is likely understanding the mobilome and discerning linkages between enterococci in wastewater and other environmental and clinical sources.

## Methods

### Isolate selection

Thirty-nine *Enterococcus spp*., including *E. faecalis* (*n* = 24), *E. faecium* (*n* = 11), *E. casseliflavus* (n = 2) and *E. gallinarum* (n = 2), isolated from wastewater were selected for whole genome sequencing. These were selected from a collection of 308 isolates from the primary and final effluents of two WWTPs in Kingston, Ontario, Canada, a BAF and a CAS system between 2014 and 2016. Isolates were speciated and subsequently underwent disc susceptibility testing for a panel of 12 antimicrobial agents. Nine to ten *Enterococcus* isolates were chosen from each of the primary and final effluent of the two WWTPs to represent the most prominent species isolated from the samples and the most prominent unique antimicrobial resistance phenotypic profiles. While all of these isolates grew in Todd-Hewitt broth supplemented with vancomycin (≥ 4 mg/L), not all met the requirements for vancomycin resistance using disc susceptibility testing following CLSI and EUCAST guidelines. This procedure used reference strains *E. faecium* ATCC 700221 (MIC ≥32 mg/L), *E. faecalis* ATCC 51299 (MIC ≥4 mg/L) and *E. faecalis* ATCC 29212 (susceptible) and *Staphylococcus aureus* ATCC 25923. The final isolates selected included 21 vancomycin-susceptible, multi-drug resistant enterococci and 18 enterococci with either intermediate resistance or resistance to vancomycin based on disc susceptibility testing. The AMR phenotypic profiles of the selected isolates are available in Table 2.

### DNA extraction and sequencing

*Enterococcus spp*. were grown on Brain Heart Infusion (BHI) agar (Dalynn Biologicals, Calgary, AB) overnight at 37 °C. Colonies from a freshly grown culture plate were suspended in TE buffer to achieve an OD_600_ of 2 in order to harvest 2 × 10^9^ cells, and 1 mL was transferred to a microcentrifuge tube and centrifuged for 2 min at 14000 x g. Genomic DNA was extracted using a modified DNeasy Blood & Tissue Kit (Qiagen, Hilden, Germany) with the addition of an enzymatic lysis step. Bacterial cells were incubated at 37 °C with shaking (150 rpm) in lysis buffer consisting of 20 mM Tris-Cl (pH 8.0), 2 mM sodium EDTA, 1.2% Triton X-100 and 40 mg/mL lysozyme (Sigma Aldrich Canada, Oakville, ON). Proteinase K and 5 μL of 100 mg/mL RNase A were added (Qiagen, Hilden, Germany), and the mixture was incubated at room temperature for 10 min before proceeding to the next step. The quality of the genomic DNA was determined using a Nanodrop One UV-Vis Spectrophotometer (Thermo Scientific, Burlington, ON) and a Qubit fluorometer (Thermo Scientific). Genomic library construction was performed using the Illumina Nextera XT DNA sample preparation kit (Illumina Inc., San Diego, CA) following the manufacturer’s instructions. The library was sequenced on an Illumina MiSeq platform (Illumina, Inc.). FASTA data was filtered for quality and high-quality reads were de novo assembled using SPAdes genome assembler 3.6.0 [4] and annotated using Prokka 1.12 ([63].

### Comparative analysis

Pangenomic analysis was completed using the contigs extracted from the Genbank file which were re-annotated using Prokka 1.13.3 (Seeman, 2014). This generated GFF files that were used as input to Roary 3.12 [46]. Multi-locus sequence typing (MLST) was performed using online MLST databases. In particular, the *Enterococcus faecalis* MLST (https://pubmlst.org/ efaecalis/) and *Enterococcus faecium* MLST (https://pubmlst.org/ efaecium/) based at the University of Oxford [30] and funded by the Wellcome Trust. The phylogenetic trees were constructed based on analysis of single nucleotide variants (SNVs) of the core genes. The phylogenetic analyses were conducted using a single nucleotide variant phylogenomics (SNVPhyl) pipeline [49] using unassembled sequence read data. The paired-end reads for Illumina sequencing of the 39 *Enterococcus spp.* genomes were aligned to the appropriate reference genome to generate read pileups (SMALT v.0.7.5; http://www.sanger.ac.uk/science/tools/smalt-0). The presence and absence matrices were generated using Phandango [23]. Whole genome sequences of *E. faecalis* and *E. faecium* (Additional file 1) were also included in the analysis and were ran through the ART next-generation sequencing read simulator [27] to generate paired-end reads with length and coverage similar to the experimental dataset (2 × 300 base PE and ~50X coverage). The reads were subject to mapping quality filtering (minimum mean mapping quality score of 30) and coverage (15X minimum coverage threshold) estimations. Using a single nucleotide variant (SNV) abundance ratio of 0.75, with no SNV density filtering setting, variant calling, variant consolidation and single nucleotide variant alignment generation of the final phylogeny was run through PhyML [22] using the maximum likelihood method. The resulting tree was visualized using interactive Tree of Life (iTOL) version 4.2.1 (https://itol.embl.de/). Assignment of proteins into clusters of orthologous groups (COGs) was performed using the compare genomes function of DOE Joint Genome Institute Integrated Microbial Genomes & Microbiomes platform [38]. Correlations were calculated using R statistical platform version 3.4.3 (R [16]) and figures were generated using packages Hmisc [25] and corrplot [76].

Draft genome sequences of the 39 *Enterococcus spp*. were investigated for the presence of putative virulence and AMR genes, mobile gene elements, bacteriophage, and CRISPR/Cas arrays. The contigs of each draft genome were ordered based on alignment against a reference genome using progressive Mauve [17]. Virulence and AMR genes were identified using Virulence Finder version 1.5 [29] and CARD version 2.0.1 [28], respectively. Results for AMR genes were further verified using megaBLAST and hits were manually curated. Genomes were investigated for integrative conjugative elements (ICEs) by homology searches using BLAST against 466 ICEs downloaded from the ICEberg database 1.0 [8]. The genomes were then analyzed for the presence of prophage using PHAST [81]. CRISPR-Cas arrays were identified using the CRISPRdb [20]. Secondary metabolite biosynthetic gene clusters were identified using the Antibiotics and Secondary Metabolite Analysis Shell (antiSMASH) version 3.0 [75].

A biomarker search was carried out with the 39 genomes from this study and an additional 59 genomes retrieved from NCBI using Neptune [37] and a Galaxy instance from the National Microbiology Laboratory in Winnipeg, MB, Canada. The inclusion and exclusion groups are listed in Additional file 1 (Sheet 19). The cut-off score for signatures among species was 95% and the cut-off score for signatures within species from different sources was 80%. The functions related to the genes covered by each signature was identified by mapping the signatures to a reference, then identifying the functions of the genes using UniProt [70]. The reference genomes that were used were *E. faecalis* V583 (NC_004668), *E. faecium* DO (NC_017960), and *E. casseliflavus* B72 (this study).

## Supplementary information

**Additional file 1: **Supplementary Details on Genomic Comparisons. Sheet 1: Complete List of Genomes Used; Sheet 2: Genome Characteristics of Sequenced Genomes; Sheet 3: COG Analysis; Sheet 4: COG Pivot Chart; Sheet 5: COG graphs; Sheet 6: COG correlation and *p* values; Sheet 7: AMR phenotypic profiles and breakpoints; Sheet 8: AMR genes detected; Sheet 9: Virulence Factors Raw Output; Sheet 10: Virulence Factors by Species; Sheet 11: Virulence Factors Summary; Sheet 12: Phage Detected; Sheet 13: CRISPRfinder Output; Sheet 14: CRISPRfinder Confirmed Systems; Sheet 15: CRISPRdb BLAST Results; Sheet 16: CRISPR Summary Table; Sheet 17: ICEberg Raw Results; Sheet 18: antiSMASH results; Sheet 19: Neptune Biomarker Combinations

## Data Availability

The genome sequences can be accessed after 2020-02-26 at https://www.ncbi.nlm.nih.gov/bioproject/browse using Bioproject PRJNA524668. Until then, the sequences are available from the corresponding author upon reasonable request.

## Abbreviations

AMR: Antimicrobial resistance
AntiSMASH: Antibiotics and Secondary Metabolite Analysis Shell
ARG: Antimicrobial resistance gene
BAF: Biological aerated filter
BHI: Blood Heart Infusion
BLAST: Basic local alignment search tool
CARD: Comprehensive Antimicrobial Resistance Database
CAS: Conventional activated sludge
CLST: Clinical and Laboratory Standards Institute
COGs: Clusters of orthologous groups
CRISPR/Cas: Clustered regularly interspaced short palindromic repeats and CRISPR-associated genes
CRISPRdb: CRISPR database
CrRNAs: CRISPR RNA
EUCAST: European Committee on Antimicrobial Resistance Testing
ICE: Integrated conjugative element
ITOL: Interactive tree of life
MGE: Mobile genetic element
PHAST: Phage search tool
SNVPhyl: Single nucleotide variants phylogenomics pipeline
ST: Sequence type
VRE: Vancomycin-resistant enterococci
WWTP: Wastewater treatment plant
